# Current and Emerging Biomarkers Predicting Bone Metastasis Development

**DOI:** 10.3389/fonc.2020.00789

**Published:** 2020-06-03

**Authors:** Michele Iuliani, Sonia Simonetti, Giulia Ribelli, Andrea Napolitano, Francesco Pantano, Bruno Vincenzi, Giuseppe Tonini, Daniele Santini

**Affiliations:** Medical Oncology, Bio-Medico University of Rome, Rome, Italy

**Keywords:** bone metastases, CTCs, DTCs, ctDNA, miRNAs, bone turnover markers

## Abstract

Bone is one of the preferential sites of distant metastases from malignant tumors, with the highest prevalence observed in breast and prostate cancers. Patients with bone metastases (BMs) may experience skeletal-related events, such as severe bone pain, pathological fractures, spinal cord compression, and hypercalcemia, with negative effects on the quality of life. In the last decades, a deeper understanding of the molecular mechanisms underlying the BM onset has been gained, leading to the development of bone-targeting agents. So far, most of the research has been focused on the pathophysiology and treatment of BM, with only relatively few studies investigating potential predictors of risk for BM development. The ability to select such “high-risk” patients could allow early identification of those most likely to benefit from interventions to prevent or delay BM. This review summarizes several evidences for the potential use of specific biomarkers able to predict early the BM development.

## Introduction

Bone is a common site for tumor metastasis, particularly for breast, prostate, kidney, and lung cancers (1). Osteotropism is defined as the stepwise process whereby tumor cells acquire specific molecular characteristics that allow them to detach from the primary tumor and spread into the bloodstream and home within the bone niche. The highly vascular nature of the bone marrow, as well as the presence of pro-angiogenic cytokines and growth factors, contribute to the establishment of a favorable soil for cancer cells seeding and surviving in premetastatic sites. Once in the bone marrow, cancer cells (known as disseminated tumor cells, DTCs) may remain dormant or lead to the development of overt BM, even after prolonged periods of latency (2–5). The presence of DTCs in the bone marrow is correlated with an increased risk of disease recurrence and poor prognosis in early breast cancer (BCa) patients (6–8). Based on these evidences, bone-targeted agents' efficacy has been tested in adjuvant setting (9). In this regard, prospective randomized controlled trials have been designed showing conflicting results (9–14). In particular, the use of adjuvant bisphosphonates was associated to a reduction in the incidence of BM, but benefits on overall survival were restricted to specific patient subgroups (10–14). Similar conflicting results were reported with adjuvant denosumab, a human monoclonal antibody that inhibits the receptor activator of nuclear factor κB ligand (RANKL). In the ABCSG-18 trial, adjuvant treatment with denosumab improved disease-free survival in patients with hormone receptor-positive BCa (15), whereas in the D-CARE trial, denosumab did not significantly increase BM-free survival in women with stage II or III BCa (16).

**Figure 1:**
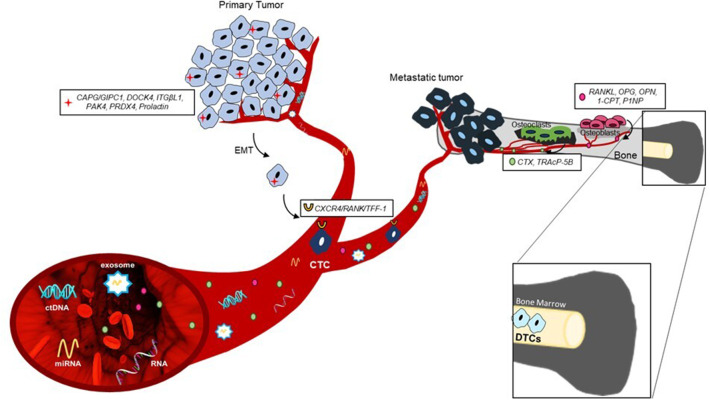
Principal predictive biomarkers of bone metastasis development.

The identification at an early stage of the disease of patients at high risk for developing BM could consequently increase the impact by a bone-specific adjuvant treatment. Here, we report preclinical and clinical evidences on promising circulating and tissue biomarkers that could be useful for the prediction or early diagnosis of BM, as summarized in Figure 1.

## Expression Profile in Primary Tumor as Biomarker for Predicting Bone Metastases

Several authors reported that protein or gene expression profiles of the primary tumor might predict later BM development (Table 1). Westbrook et al. reported that the composite expression of the two proteins macrophage-capping protein (CAPG) and GIPC PDZ domain-containing protein (GIPC1) in primary BCa tissues of patients enrolled in the phase III AZURE trial strongly predicted skeletal disease-free survival (DFS) and overall survival (OS) (17). Interestingly, adjuvant zoledronate treatment significantly reduced distant bone recurrence only in patients with high expression of both proteins (17). These data suggest that CAPG and GIPC1 expression in primary BCa tissue might be both prognostic and predictive of efficacy with adjuvant zoledronate treatment. Xiao-Qing Li et al. identified integrin beta-like 1 (ITGBL1) as a candidate biomarker predicting BM development. Indeed, ITGBL1 was coexpressed with genes related to osteomimicry in primary BCa tissues and correlated with BM occurrence (18).

**Table 1:** Expression gene profiles predicting bone metastases.

**Biomarker**	**Tumor type**	**Predictive role in bone metastasis**	**References**
CAPG, GIPC1	Breast cancer	High expression levels	(17)
ITGBL1	Breast cancer	Coexpressed with other genes related to osteomimicry	(18)
IL-1 β	Breast cancer	High expression levels	(19)
DOCK-4	Breast cancer	High levels of expression	(20)
nPAK4	Breast cancer	Elevated gene expression	(21)
PRDX4, LPC1	Breast, prostate, and renal cancers	High levels of expression	(22)
PRL, PRLR	Breast cancer	Elevated gene expression	(23–25)
GESBN model	Breast cancer	A panel of 51 genes predict bone recurrence	(26, 27)

Moreover, gene expression and proteomics analysis on BCa cells more prone to cause BM in xenograft murine models might also help in the identification of relevant biomarkers. For example, interleukin (IL)-1β was found to be upregulated in a bone-seeking model of BCa cells, and further investigation on 150 primary BCa core biopsies showed a significant correlation between its expression and BM onset (19). Importantly, Holen et al. demonstrated the efficacy of IL-1β inhibitors in preventing skeletal events in experimental mouse models (28). In a similar model, the dedicator of cytokinesis protein 4 (DOCK4) was also identified as another potential biomarker of BM. This preclinical result was also validated by tissue microarray from the large AZURE adjuvant study (20). In the control group, higher DOCK4 expression was significantly prognostic for first bone distant recurrence, whereas in the zoledronic group, this association was lost, suggesting that treatment with zoledronate may counteract the higher risk for bone relapse from high DOCK4-expressing tumors (20). Importantly, DOCK4 expression was not associated with risk of non-skeletal events (20).

Additional candidate biomarkers have been recently identified as predictors of metastatic spread to the bone: among these, nuclear p21-activated kinase 4 (nPAK4) expression was associated with BM development specifically in estrogen receptor alpha (ERα) positive BCa patients via targeting of the leukemia inhibitory factor receptor (LIFR), a BM suppressor (21). Other osteoclastogenesis mediators, including peroxiredoxin-4 (PRDX4) and L-plastin (LPC1), have been identified as responsible for tumor bone colonization in a number of osteotropic cancers such as breast, prostate, and renal cancers (22). Furthermore, an association between increased levels of circulating prolactin (PRL) and BCa metastases has been reported (23, 24), and recent studies showed that high expression of the PRL receptor (PRLR) on a primary tumor correlated with a shorter time to BM (25).

Recently, Li et al. (26) identified a panel of 51 genes differentially expressed between non-metastatic and bone metastatic BCa patients, starting from a merged data set containing clinical and transcriptomic data of 855 BCa patients. The panel validated by survival analyses showed a high performance in predicting BM. Similarly, Zhao et al. (27) developed a gene expression signature-based nomogram model to predict BM in BCa patients. In particular, using three microarray data sets of 572 patients, including 191 with BM and 381 metastases-free, they identified five BM-related genes: keratin 23 (KRT23), receptor accessory protein 1 (REEP1), spi-B transcription factor (SPIB), aldehyde dehydrogenase 3 family member B2 (ALDH3B2), and glycine decarboxylase (GLDC). These genes were then used to set up a model able to identify bone recurrence with high predictive power (with a C-index of 0.677 for the training set and 0.689 and 0.695 for the testing sets, respectively).

Although this and other models could represent useful prediction tools for the clinicians, most of the biomarkers derived from protein and gene expression profiles do not currently have standardized analytical tools to be measured and therefore have not been sufficiently validated to be widely adopted.

## Role of Circulating Tumor Cells and Disseminated Tumor Cells in Predicting Bone Recurrence

Circulating tumor cells (CTCs) are defined as cancer cells originating from primary and/or metastatic sites and circulating in the bloodstream. CTCs have shown prognostic implications in a variety of cancer types, including BCa, prostate cancer (PCa), non-small cell lung cancer (NSCLC), colorectal cancer, and others (29). CTCs provide clinical relevant information about tumor burden, biological aggressiveness of the disease, the presence of undetectable micrometastases, and the tendency to metastatic spread.

Several evidences suggest that CTCs count can be used as an early predictor of bone metastatic potential in PCa (30), BCa (31), and NSCLC (32) (Table 2). In particular, in castration-resistant prostate cancer (CRPC) patients, CTC detection was closely associated with the clinical evidence of BM and with survival (30). Similarly, a higher CTC number were detected in patients with BCa with BM relative to those with no bone lesions, and in patients with multiple bone metastases relative to those with one or two bone lesions (31). Higher baseline CTC count was also predictive of BM development in lung cancer (LC) patients (32).

**Table 2:** Predictive role of CTCs and DTCs in bone metastasis onset.

**Biomarker**	**Tumor type**	**Predictive role in bone metastasis**	**References**
CTCs	Prostate, breast, and lung cancers	High CTC count	(30–32)
CTCs	Breast cancer	TFF-1 expression on CTCs	(33)
CTCs	Breast cancer	RANK expression on CTCs	(34)
CTCs	Neuroendocrine tumors	CXCR4 expression on CTCs	(35)
DTCs	Breast cancer	High DTC count	(36)
DTCs	Breast cancer	Postoperative presence of DTCs	(7)
DTCs	Prostate cancer	DTC presence at baseline	(37, 38)

In addition, the molecular characterization of CTCs may carry relevant biological information regarding the heterogeneity of the metastatic disease. Wang et al. identified a gene profile in circulating BCa cells significantly associated with BM presence. This signature showed that trefoil factor 1 (TFF1) was the most correlated gene with BM onset (39). Another study reported a strong association in the expression of several genes related to disease progression and therapy resistance between CTCs and bone metastatic tissue of PCa patients (33).

These evidences support a potential role of CTC phenotyping as a tool to predict BM onset. In this regard, we recently identified a receptor activator of nuclear-factor–κB (RANK)-positive CTC in bone metastatic BCa patients, suggesting that RANK expression may represent a phenotypic and biologic property of cancer cells with elevated osteotropism (34). This is further supported by the evidence of a strong correlation between high RANK expression in BCa as well as other primary tumor types and BM relapse (40, 41).

CTC presence is associated with BM also in patients affected by neuroendocrine tumors (NETs) (35). Interestingly, in these patients, a high percentage of CTCs expressed C-X-C chemokine receptor 4 (CXCR4), a well-known molecule involved in osteotropism (35).

Besides CTCs, several evidences have shown an association between presence of DTCs and BM occurrence in stage IV BCa. Moreover, a higher frequency of DTCs was observed in patients with lobular carcinoma, the histotype that most frequently spread to bone, compared with ductal carcinoma (36). A pooled prospective analysis of more than 4,000 BCa patients demonstrated that DTC identification in bone marrow predicted postoperative disease recurrence, including BM (7). Similarly, DTC count in bone marrow aspirates of PCa patients, collected before the initiation of primary therapy, was an independent prognostic factor of patients' survival and bone relapse (37, 42). It is well-established that the persistence of DTCs during follow-up is associated with a shorter relapse-free survival and poorer prognosis (38, 43). Interestingly, the presence of DTCs in the bone marrow is a predictor of bone-specific recurrence and could be used to identify patients with high risk to develop skeletal disease (Table 2).

## Circulating Tumor DNA and Mirnas as Non-Invasive Biomarker for Bone Metastases Prediction

In the last few years, several studies demonstrated the potential clinical utility of circulating tumor DNA (ctDNA) both in the early diagnosis of tumors and in the monitoring of therapeutic efficacy. ctDNA contains tumor-specific genetic and epigenetic alterations, which makes it a useful non-invasive prognostic and predictive biomarker in different solid tumors (44). A number of studies support the idea that ctDNA levels might be predictors of BM development (Table 3). In particular, the presence of ctDNA at baseline was associated with BM in newly diagnosed patients with advanced NSCLC (45). Similar results were obtained in late-stage NSCLC patients in which higher levels of ctDNA were associated to BM presence (46). A recent study demonstrated that MET alterations detected in ctDNA correlated with BM affected by different solid tumors (47). Since MET is greatly expressed in the bone microenvironment (53), it is therefore conceivable that the high rates of ctDNA bearing MET alterations derive from secondary bone lesions. Therefore, ctDNA profiling could represent an excellent tool to detect these specific alterations and anticipate bone metastatic recurrence prior to clinical detection.

**Table 3:** Predictive role of ctDNA and miRNA in bone metastasis onset.

**Biomarker**	**Tumor type**	**Predictive role in bone metastasis**	**References**
ctDNA	Lung cancer	Presence of ctDNA at baseline	(45)
ctDNA	Lung cancer	Higher ctDNA levels	(46)
ctDNA	Gastrointestinal, brain, lung, breast, and others	Presence of MET alterations	(47)
miRNA	Breast cancer	miR-19a, miR-93, miR-106a score	(48)
miRNA	Hepatocellular carcinoma	miR-34a reduced serum levels	(49)
miRNA	Breast cancer	miR-30 family low expression in primary tissue	(50)
miRNA	Prostate cancer	miR-466 low expression	(51)
miRNA	Breast cancer	miR-135 and miR-203 absence in metastatic tissues	(52)

MicroRNAs (miRNAs) are endogenous non-coding small RNAs that play a key role in various biological processes including bone remodeling (54, 55). Thanks to their high stability in blood, miRNAs have become promising biomarker candidates for cancer detection and monitoring, predicting outcomes and chemoresistance. Several evidences have shown a possible role of miRNAs as novel specific biomarkers of bone recurrence (Table 3). Recently, a three-miRNA signature score, which includes miR-19a, miR-93, and miR-106a, has been identified as a predictor of BM occurrence in BCa using The Cancer Genome Atlas (TCGA) datasets (48). It would be important to validate their expression levels in early BCa patients to assess their ability to predict the BM onset.

A miRNA microarray analysis in hepatocellular carcinoma (HCC) patients, with and without skeletal disease, showed that serum miR-34a expression levels were independent predictors of BM development (49). Previous evidences reported a critical role of miR-34a as a suppressor of osteoclastogenesis and bone resorption through the targeting of transforming growth factor-β-induced factor 2 (Tgif2) (56).

Recently, Croset et al. demonstrated a direct involvement of miR-30 family members in promoting BCa BM *in vitro* and *in vivo*. In addition, they found that low expression of miR-30 in primary tumors was correlated with poor relapse-free survival (50). Serum analyses of miR-30 members in a prospective trial of non-metastatic BCa patients could give a further confirmation of their predictive value in the early detection of BM.

The microRNA miR-466 has been significantly associated with BM development in PCa (51). In xenograft models, miR-466 overexpression interrupts runt-related transcription factor 2 (RUNX2) integrated network of genes preventing BM. In addition, miR-466 expression in primary tissue also predicted biochemical relapse, suggesting its clinical significance in bone metastatic process (51). The other two RUNX2-targeting microRNAs, miR-135 and miR-203, were associated to BCa growth in bone (52). In particular, these miRNAs were absent in BM expressing high levels of RUNX2, suggesting their fundamental role in regulating tumor osteotropism mediated by RUNX2 (52). Since RUNX2 represents a key player of bone metastatic process, the detection of RUNX2-targeting microRNAs in the blood could be extremely useful to monitor and control skeletal disease progression.

More recently, exosomal miRNAs have emerged as important regulators of BM in preclinical studies (57). It is well-established that tumor-derived exosomes can affect bone remodeling promoting the vicious cycle of BM (58). So far, only a few studies reported a correlation between specific exosomal miRNAs and bone metastases. Valencia et al. demonstrated that exosomes carrying miR-192 reduced metastatic bone colonization (59); on the contrary, Hashimoto et al. found high levels of specific miRNAs in exosomes of PCa cells with elevated propensity to metastasize into the skeleton (60). Considering the accumulating evidences regarding the role of exosomal miRNAs in cancer, this area of investigation should be further developed.

## Changes in Biochemical Markers of Bone Turnover Predict Bone Metastases

Biochemical markers of bone metabolism reflect the bone turnover, and variations in their levels have been correlated with BM onset and their complications (61, 62) (Table 4). The determination of bone markers in the serum and/or urine could provide a non-invasive procedure that is helpful in predicting and monitoring the progression of disease into the skeleton. Alteration of these markers reflects specific changes in bone microenvironment, which becomes a fertile niche for tumor cell homing.

**Table 4:** Bone turnover markers predicting bone relapse.

**Biomarker**	**Tumor type**	**Predictive role in bone metastasis**	**References**
P1NP, CTX and 1-CTP	Breast cancer	High serum levels	(63)
CTX	Breast cancer	High serum levels	(64)
Vitamin D	Breast cancer	Vit D deficiency	(65)
P1NP	Prostate cancer	High serum levels	(66)
TRAcP-5b	Prostate and Breast cancers	High serum levels	(67, 68)
OPG/RANKL	Prostate cancer	Alteration of OPG/RANKL balance	(69, 70)
Osteopontin	Renal carcinoma	High serum levels	(71, 72)
NTX, P1NP, CTX, 1-CTP, TRAcP-5b	Lung cancer	High serum levels	(73–78)

Patients with high serum levels of N-terminal propeptide of type-1 collagen (P1NP), C-telopeptide of type-1 collagen (CTX), and pyridinoline cross-linked carboxy-terminal telopeptide of type-1 collagen (1-CTP) after diagnosis were shown to be at high risk for bone recurrence, but not for other metastatic sites. In addition, none of these markers was predictive of treatment benefit from zoledronic acid (63).

Moreover, in the NCIC CTG MA.14 study, a high CTX serum level correlated with bone-only relapse probably due to an increased bone metabolism that may facilitate the development of skeletal metastasis (64). Conversely, any correlation between high CTX-I and P1NP levels and bone relapse was found (65), but, surprisingly, normal levels of serum vitamin D were associated with a lower risk of BM occurrence.

Several studies have reported strong correlations between elevated levels of bone turnover markers (BTMs) and the presence and the extent of skeletal disease in PCa (79, 80). Interestingly, increased P1NP levels identified PCa patients with BM vs. lymph node metastases before the first positive bone scintigraphy (66). Other studies identified significant associations between elevated plasma levels of tartrate-resistant acid phosphatase 5b (TRAcP-5b) (67, 68), osteoprotegerin (OPG) (69, 70), and osteopontin, and presence of BM in PCa and renal cancer patients (71, 72). Similarly, serum levels of BTM [such as N-terminal telopeptide (NTX), CTX, TRAcP-5b, P1NP] are strongly associated with the development and progression of BM in patients with LC (73–78).

Overall, these evidences highlighted the potential role of BTM as predictors of BM occurrence in different solid tumors.

## Discussion

The identification of patients at risk for BM could offer the opportunity to treat them at an earlier stage, improving their clinical outcomes.

In the last decades, genomic and proteomic analyses have led to the identification of molecular signatures on tumor tissue that predict bone relapse with sufficient accuracy. Indeed, several tissue biomarkers have been identified as predictive for BM development, including the composite CAPG/GIPC1 proteins and DOCK4, with the latter clinically validated. In addition, the emerging use of computational models to generate predictive signatures has significantly grown in the last years thanks to the availability of high-throughput datasets and novel data analysis tools.

More recently, liquid biopsy has emerged as a rapid, noninvasive source of biomarkers including CTCs, DTCs, ctDNA, and circulating miRNA. Liquid biopsy has the strong advantage to overcome tumor heterogeneity and capture the changing and evolving landscape of cancer in real time during the course of the disease. The molecular characterization of CTCs showed that the expression of osteotropic markers such as RANK and CXCR4 could be responsible for tumor cell homing to the bone. Thus, CTC phenotyping could dynamically track changes in tumor cell profile and predict their migration into the skeleton. Several procedures have been developed in the last decades for CTC isolation and detection, but so far the Food and Drug Administration has approved CellSearch (CS) as the unique platform for CTC enumeration. Nevertheless, CTC identification by CS based on biological characteristics (e.g., the expression of the epithelial markers such as epithelial cell adhesion molecule, EpCAM, and cytokeratins) does not reach 100% of sensitivity and specificity. For example, patients with epithelial cancers might present CTCs expressing mesenchymal rather than epithelial markers, as a result of epithelial-to-mesenchymal transition, a phenomenon associated to disease progression (81–83). These technical limitations have slowed the diagnostic and prognostic use of CTC blood test into clinical practice. DTCs have been demonstrated to be strong predictors of BM onset in both early BCa and PCa. Similar to CTCs, also DTC detection and analysis present some technical limitations including a low number of cells and the difficulty to characterize them with standard technologies such as flow cytometry, immunofluorescence, or polymerase chain reaction (PCR). Moreover, BM aspiration procedure is an invasive method that cannot be repeated unlimitedly.

Since the release of ctDNA into the bloodstream is frequently in cancer patients, screening of ctDNA may provide clinically relevant information about mutational profiles associated with BM development. There are still many challenges that need to be overcome before its introduction in clinical practice. Due to the extremely low levels in the blood, ctDNA sensitivity and specificity remain the principal issues. Current digital PCR methods fail to detect smaller fragments derived from tumors increasing false negative, but advances in genomic approaches could allow us to identify all ctDNA in the blood. Due to their high stability in the blood, circulating miRNAs are probably the most promising biomarkers of bone recurrence. Indeed, several miRNAs have been identified as key regulators of the principal genes involved in bone remodeling and cancer bone tropism. The development of different technical platforms over other RNA-seq technologies guarantees an intrinsic technical reproducibility needed for their rapid translation in clinical practice.

Finally, BTM could represent easily measured factors that are able to predict BM in patients with early stage of cancer. Indeed, P1NP, CTX, and 1-CTP were found to be predictive of bone-specific recurrence, suggesting that an increased bone turnover creates a fertile environment that promotes cancer cell adhesion and growth. Nevertheless, BTM levels can be influenced not only by patients' features, such as age, sex, and food intake, but also by systemic treatments that affect bone remodeling.

## Author Contributions

DS and MI contributed conception and design of the study. SS and MI wrote the first draft of the manuscript. GR, SS, and FP performed figure and tables of the manuscript. DS, GT, BV, and FP revised the manuscript. All authors contributed to read and approved the submitted version.

## Conflict of Interest

The authors declare that the research was conducted in the absence of any commercial or financial relationships that could be construed as a potential conflict of interest.

## Abbreviations

ALDH3B2: Aldehyde dehydrogenase 3 family member B2
BCa: breast cancer
BM: bone metastases
BTM: bone turnover markers
CTX: C-telopeptide of type-1 collagen
CXCR4: C-X-C chemokine receptor 4
CRPC: castration-resistant prostate cancer
CS: CellSearch
CTCs: circulating tumor cells
ctDNA: circulating tumor DNA
DOCK4: dedicator of cytokinesis protein 4
DTCs: disseminated tumor cells
EpCAM: epithelial cell adhesion molecule
EMT: epithelial–mesenchymal transition
ERα: estrogen receptor alpha
GIPC1: GIPC PDZ domain containing family, member 1
GLDC: glycine decarboxylase
HCC: hepatocellular carcinoma
ITGBL1: integrin beta-like 1
IL-1β: interleukin-1β
KRT23: keratin 23
LPC1: L-plastin
LIFR: leukemia inhibitory factor receptor
LC: lung cancer
CAPG: macrophage-capping protein
miRNAs: microRNAs
P1NP: N-terminal propeptide of type-1 collagen
NTX: N-terminal telopeptide
NET: neuroendocrine tumors
NSCLC: non-small cell lung cancer
nPAK4: nuclear p21-activated kinase 4
RANK: nuclear-factor–κB
OPG: osteoprotegerin
OS: overall survival
PRDX4: peroxiredoxin-4
PCR: polymerase chain reaction
PRLR: PRL receptor
PRL: prolactin
PCa: prostate cancer
1-CTP: pyridinoline cross-linked carboxy-terminal telopeptide of type-1 collagen
REEP1: receptor accessory protein 1
RANKL: receptor activator of nuclear factor κB ligand
RUNX2: runt-related transcription factor 2
GESBN: signature-based nomogram
SPIB: Spi-B transcription factor
TRAcP-5b: tartrate-resistant acid phosphatase 5b
TCGA: The Cancer Genome Atlas
TMA: tissue microarray
Tgif2: transforming growth factor-β-induced factor 2
TFF1: Trefoil factor 1.
